# Definition of joint biomarker signatures for patients with locally advanced HNSCC treated by postoperative radiochemotherapy – A multicentre study of the DKTK-ROG

**DOI:** 10.1016/j.ctro.2026.101330

**Published:** 2026-10-25

**Authors:** Steffen Löck, Annett Linge, Fabian Lohaus, Stefan Schmidt, Max Kemper, Dominik Haim, Inge Tinhofer, Goda Kalinauskaite, Ali Sak, Maja Guberina, Martin Stuschke, Panagiotis Balermpas, Franz Rödel, Claus Rödel, Michael Henke, Henning Schäfer, Anca-Ligia Grosu, Max Knoll, Bouchra Tawk, Amir Abdollahi, Jürgen Debus, Julia Hess, Kristian Unger, Claus Belka, Steffi Pigorsch, Stephanie E. Combs, Chiara De-Colle, Simon Boeke, Daniel Zips, Gustavo B. Baretton, Mechthild Krause, Michael Baumann

**Affiliations:** a German Cancer Consortium (DKTK), Partner Site Dresden, and German Cancer Research Center (DKFZ), Heidelberg, Germany; b German Cancer Consortium (DKTK), Partner Site Berlin, and German Cancer Research Center (DKFZ), Heidelberg, Germany; c German Cancer Consortium (DKTK), Partner Site Essen, and German Cancer Research Center (DKFZ), Heidelberg, Germany; d German Cancer Consortium (DKTK), Partner Site Frankfurt, and German Cancer Research Center (DKFZ), Heidelberg, Germany; e German Cancer Consortium (DKTK), Partner Site Freiburg, and German Cancer Research Center (DKFZ), Heidelberg, Germany; f German Cancer Consortium (DKTK), Partner Site Heidelberg, and German Cancer Research Center (DKFZ), Heidelberg, Germany; g German Cancer Consortium (DKTK), Partner Site Munich, and German Cancer Research Center (DKFZ), Heidelberg, Germany; h German Cancer Consortium (DKTK), Partner Site Tübingen, and German Cancer Research Center (DKFZ), Heidelberg, Germany; i OncoRay – National Center for Radiation Research in Oncology, Faculty of Medicine and University Hospital Carl Gustav Carus, TUD Dresden University of Technology, Helmholtz-Zentrum Dresden-Rossendorf, Dresden, Germany; j Helmholtz-Zentrum Dresden-Rossendorf, Institute of Radiooncology – OncoRay, Dresden, Germany; k National Center for Tumor Diseases (NCT), NCT/UCC Dresden, a partnership between DKFZ, Faculty of Medicine and University Hospital Carl Gustav Carus, TUD Dresden University of Technology, and Helmholtz-Zentrum Dresden-Rossendorf (HZDR), Germany; l Department of Radiotherapy and Radiation Oncology, Faculty of Medicine and University Hospital Carl Gustav Carus, TUD Dresden University of Technology, Dresden, Germany; m Department of Otorhinolaryngology, Faculty of Medicine and University Hospital Carl Gustav Carus, Technische Universität Dresden, Germany; n Department of Oral and Maxillofacial Surgery, Faculty of Medicine and University Hospital Carl Gustav Carus, Technische Universität Dresden, Germany; o Department of Radiooncology and Radiotherapy, Charité, University Medicine Berlin, Germany; p Department of Radiotherapy, Medical Faculty, University of Duisburg-Essen, Essen, Germany; q Department of Radiotherapy and Oncology, Goethe-University Frankfurt, Germany; r Department of Radiation Oncology, Medical Center, Medical Faculty, University of Freiburg, Germany; s Heidelberg Institute of Radiation Oncology (HIRO), National Center for Radiation Research in Oncology (NCRO), University of Heidelberg Medical School and German Cancer Research Center (DKFZ), Germany; t Heidelberg Ion Therapy Center (HIT), Department of Radiation Oncology, University of Heidelberg Medical School, Germany; u National Center for Tumor Diseases (NCT), University of Heidelberg Medical School and German Cancer Research Center (DKFZ), Germany; v Clinical Cooperation Unit Radiation Oncology, University of Heidelberg Medical School and German Cancer Research Center (DKFZ), Germany; w Research Unit Translational Metabolic Oncology (TMO), Institute for Diabetes and Cancer (IDC), Helmholtz Diabetes Center, Helmholtz Munich, Neuherberg, Germany; x Clinical Cooperation Group Personalized Radiotherapy in Head and Neck Cancer, Helmholtz Munich, Neuherberg, Germany; y Joint Heidelberg-IDC Translational Diabetes Program, Department of Inner Medicine I, Heidelberg University Hospital, Heidelberg, Germany; z Department of Radiation Oncology, LMU University Hospital, Ludwig Maximilians University Munich, Munich, Germany; aa German Center for Diabetes Research (DZD), Neuherberg, Germany; ab Bavarian Center for Cancer Research, Munich, Germany; ac Comprehensive Cancer Center, Munich, Germany; ad Department of RadioOncology, Technische Universität München, Germany; ae Department of Radiation Sciences (DRS), Institut für Innovative Radiotherapie (iRT), Helmholtz Zentrum Munich, Neuherberg, Germany; af Department of Radiation Oncology, University Hospital Tübingen, Tübingen, Germany; ag Department of Advanced Radiation Oncology, IRCCS Sacro Cuore Don Calabria Hospital, Cancer Care Center, Negrar di Valpolicella, VR, Italy; ah Institute of Pathology, Faculty of Medicine and University Hospital Carl Gustav Carus, Technische Universität Dresden, Germany; ai Tumour- and Normal Tissue Bank, University Cancer Centre (UCC), University Hospital Carl Gustav Carus, Technische Universität Dresden, Germany; aj German Cancer Research Center (DKFZ), Division of Radiooncology/Radiobiology, Heidelberg, Germany

**Keywords:** HNSCC, biomarkers, postoperative radiochemotherapy, validation, signature

## Abstract

Several prognostic biomarkers have previously been identified for patients with locally advanced head and neck squamous cell carcinoma (HNSCC) treated with postoperative radiochemotherapy (PORT-C) in a comprehensive biomarker program conducted by the German Cancer Consortium - Radiation Oncology Group (DKTK-ROG). Here, we combined these biomarkers into composite signatures for loco-regional control (LRC) and overall survival (OS) in 143 patients with HPV16 DNA-negative tumours. The C-indices for baseline, extended and data-driven signatures were 0.77, 0.93 and 0.94 for LRC and 0.69, 0.83 and 0.82 for OS, respectively. These predefined models will next be tested in the prospective HNprädBio validation cohort, which has completed recruitment and data collection.

## Highlights

• Previously published biomarkers were combined to three prognostic signatures.
• Extended signatures included additional biomarkers related to hypoxia, radiation response, miRNA expression and SNPs.
• Corresponding models are predefined for prospective validation in the HNprädBio study.

## 1 Introduction

The heterogeneous response of patients with locally advanced head and neck squamous cell carcinomas (HNSCC) to postoperative radiochemotherapy (PORT-C) necessitates the development of biomarkers to further personalise treatment in the future [1]. Such biomarkers need to be robustly developed and externally validated, optimally in prospective studies [2]. A combination of independent biomarkers may thereby strengthen prognostic power and enhance the probability of successful validation [3].

This aim is pursued by a comprehensive biomarker program, conducted by the German Cancer Consortium - Radiation Oncology Group (DKTK-ROG). The biomarker program follows three phases: (i) retrospective biomarker discovery and model development; (ii) prospective validation in the HNprädBio study; and (iii), contingent on successful validation, biomarker-guided testing in interventional clinical trials. This design is utilized for patients with HNSCC treated with primary radiochemotherapy (RCTx) and with PORT-C. In phase (i), retrospectively collected tumour specimens were analysed to identify potential prognostic biomarkers [1], [4]. For the primary RCTx cohort, these individual biomarkers were recently combined in multivariable models that are currently validated in phase (ii) of the program [5]. For the PORT-C cohort, promising biomarker candidates identified in phase (i) include cancer stem cell-associated markers, hypoxia classifiers, single-nucleotide polymorphisms, chemokines, tumour infiltrating lymphocytes, gene-expression classifiers, DNA-methylome classifiers and a miRNA classifier [1], [6], [7], [8], [9], [10], [11], [12], [13], [14], [15]. The present analysis finalizes the model definitions (i) before analysis of the prospective validation cohort (ii).

In the present manuscript, we review and compare the biomarkers that were identified for patients in the retrospective HNSCC cohort of the DKTK-ROG treated with PORT-C. Furthermore, we pre-hoc define composite signatures and corresponding prognostic models for the upcoming analysis of the prospective validation trial (HNprädBio study, NCT02059668, www.clinicaltrials.gov).

## 2 Materials and methods

### 2.1 Patient cohort

The retrospective cohort of patients with locally advanced HNSCC treated with PORT-C within the DKTK-ROG includes 221 patients with tumours originating from the oral cavity, oropharynx, or hypopharynx [1], [11]. Patients received cisplatin-based PORT-C between 2005 and 2012 with a median dose of 64.0 Gy (56.0–68.4 Gy) according to standard protocols. Data were centrally collected in the RadPlanBio platform of the DKTK [16] and reviewed at the DKTK partner site Dresden. For the present study, patients with HPV16 DNA-positive tumours were excluded, due to their extremely low number of loco-regional recurrences and their overall favourable outcome [1], resulting in 143 remaining patients.

### 2.2 Preparation of biomaterials and biomarker analyses

Biomaterial was extracted from formalin-fixed paraffin-embedded (FFPE) tissues centrally at the DKTK partner site Dresden, processed under standardized procedures and used for biomarker investigation at the DKTK-ROG sites depending on their expertise, as described previously [1], [11]. Different biomarkers were assessed, as detailed below and in Supplementary Table 1. For the present analysis, no new wet-lab biomarker experiments were performed, previously generated patient-level biomarker measurements were combined. Assay-specific technical controls and quality-control procedures are described in the cited original studies. Short summaries are given below.

Genomic DNA and total RNA were extracted from 5 μm FFPE tissue sections, as described in [1], [11]. nanoString Elements technology (nanoString Technologies, Seattle, WA, USA) was used to assess gene expressions. A hypothesis-driven gene panel was used, including the potential CSC marker *SLC3A2* as well as the 15- and 26-gene hypoxia-associated signatures [17], [18], [19]. Classification into tumours with lower or higher expression of the hypoxia-associated gene signatures was performed by k-means clustering with two cluster centres. Based on the comparison of several machine-learning and feature-selection algorithms, a 7-gene signature was developed consisting of the three individual genes *HILPDA*, *CD24*, *TCF3*, and one metagene combining the highly correlated genes *SERPINE1*, *INHBA*, *P4HA2*, and *ACTN1* [13]. Real-time PCR was performed as described previously [11] for 39 genes including the cancer stem cell-associated marker *CD44*.

The expressions of CD8 and PD-L1 were analysed in different compartments of the tumour (intraepithelial, periphery, stroma) after immunohistochemical staining of FFPE sections. The staining density was scored as negative (1), moderate (2), abundant (3), or highly abundant (4) for each compartment. A sum score ≥ 6 was considered as high expression [6].

Tissue sections were stained for SDF-1 and CXCR4 using immunofluorescence, as reported in [8]. The staining intensity in the tumour areas was scored as negative (0), low (1), intermediate (2), or high (3) in one section per patient. A staining of intensity ≥2 in at least one core was considered as positive. Staining was analysed for membrane (m) and intracellular (ic) region.

SNPs of genes related to DNA double-strand breaks and nucleotide excision repair were analysed after DNA extraction from FFPE samples using allele-specific color-coded hybridisation probes (TaqMan, Life Technologies) [9]. The following SNPs were considered: rs11615, rs13181, rs17655, rs25487, rs2267437, rs4988023.

DNA methylation profiling was performed using Illumina microarrays (Illumina, San Diego, California) as previously described [14]. An HPV-independent classifier of disease recurrence (HICR) was developed using a machine-learning framework including feature selection and bootstrapped LASSO regression. An optimal risk cutoff was selected to classify tumours into low- and high-risk groups. In addition, Hypoxia-M, a DNA methylation-based signature of hypoxia [20], [21] and PD-L1-M, a signature of PD-L1 positivity (Knoll, manuscript under preparation) were integrated in the analysis.

Total RNA, including the small RNA fraction, was extracted using the Qiagen miRNeasy FFPE Kit. miRNA expression was profiled using SurePrint G3 8 × 60 K Human miRNA Microarrays (AMADID 70156; Agilent Technologies) [10]. Based on iterative forward selection with repeated random training and validation splits, a Cox proportional hazards model for freedom from recurrence time was generated using hsa-let-7g-3p, hsa-miR-6508-5p, hsa-miR-210-5p, hsa-miR-4306, and hsa-miR-7161-3p, and the final model size was selected by Akaike information criterion. The linear predictor of the final Cox regression model constitutes the patient-wise risk score. The median risk score of the discovery cohort was used as a cutoff for risk classification.

### 2.3 Clinical endpoints, data processing and statistics

Loco-regional tumour control (LRC) was studied as the primary and overall survival (OS) as the secondary endpoint, measured from the first day of radiotherapy until the respective event occurred or the patient was censored. Survival curves were estimated by the Kaplan-Meier method. The impact of prognostic parameters on the endpoints was evaluated using Cox proportional hazards regression. Correlations were estimated using the Spearman correlation coefficient ρ.

Univariable Cox regression was performed for both endpoints. Significant features were considered for subsequent multivariable analyses using three different approaches similar to [5]. First, a baseline model was developed that included relevant clinical parameters, *CD44* gene expression as a cancer stem cell-associated marker, and hypoxia classification based on the 15-gene hypoxia-associated signature [17], [18], all of which are well-established radiobiological markers for local tumour control. In a second step, this baseline model was extended by including relevant additional biomarkers using a forward selection strategy with a *p*-value of 0.1 for inclusion and exclusion based on the likelihood ratio (extended model). The third strategy used the same forward selection procedure based on all biomarkers without fixing the parameters of the baseline signature (data-driven model). This procedure was performed for the endpoints LRC and OS individually. The concordance index (C-Index; 0.5: random prediction, 1.0: perfect prediction) was evaluated for every model and its 95% confidence interval was estimated by 1000 bootstrap samples. For multivariable modelling, missing values of the biomarkers were imputed by their median (continuous variables) or most frequent value (categorical variables). Missing values occurred typically due to limited tissue availability (Supplementary Table 1). Based on the linear predictor of the Cox models, the 67th percentile was used to define an approximately one-third high-risk group. This cut-off was chosen to retain a sufficiently sized high-risk subgroup in view of the limited number of events and was not optimized with respect to the observed survival differences. LRC and OS were compared between the risk groups using the log-rank test. Statistical analyses were performed in SPSS 30 (IBM Corporation, Armonk, NY, USA). Two-sided tests were applied and *p*-values below 0.05 were considered as statistically significant.

## 3 Results

Univariable associations of clinical parameters and biomarkers with LRC and OS are reported in Supplementary Table 2. Significant associations with LRC were observed for age, tumour location, T stage, the cancer stem cell-associated marker *CD44*, the 15- and 26-gene hypoxia-associated classifiers, icSDF-1, and for the three biomarker signatures (HICR, 7-gene, 5-miRNA). For OS, the ECE status and the methylation-based PD-L1-M and Hypoxia-M showed statistical significance, while *CD44* as well as the marker icSDF-1 did not. CD8 was associated with OS, whereas PD-L1 immunohistochemical staining did not reach statistical significance.

The Spearman correlation coefficients ρ between the different biomarkers were generally small. Moderate correlations with |ρ| ≥ 0.4 (all significantly different from zero) were observed between the 7-gene signature and tumour location in the oral cavity (ρ = 0.42), the 15-gene and 26-gene hypoxia signatures (ρ > 0.5), and HICR (ρ = 0.40); between the 26-gene hypoxia-associated signature and *CD44* expression (ρ = −0.44); and between the 5-miRNA signature and ECE status (ρ = 0.40) and HICR (ρ = 0.41). Supplementary Fig. 1 presents the correlations of selected biomarkers. The overall low correlations reflect the limited redundancy of the biomarkers, which may allow for improving endpoint prediction by biomarker combination.

For the upcoming validation of biomarkers in the currently still blinded prospective HNSCC cohort of the DKTK-ROG treated with PORT-C (HNprädBio study), we first defined a baseline biomarker signature including significant clinical parameters as well as expression of cancer stem cell-associated markers and hypoxia-classifiers, which are widely explored prognosticators for radiosensitivity in preclinical studies and for outcome in patients with locally advanced HNSCC in clinical studies [17], [18], [22], [23], [24]. Based on the univariable Cox regression results (Table 1), the baseline signature for LRC contained age, tumour location, T stage, *CD44* gene expression and the 15-gene hypoxia-associated classifier, while the baseline signature for OS contained tumour location, ECE status, T stage and the 15-gene hypoxia classifier, Table 1. The C-Index of these signatures was 0.77 (95% confidence interval: 0.67–0.86) for LRC and 0.69 (0.62–0.76) for OS.

**Table 1:** Multivariable Cox regression of loco-regional tumour control and overall survival for 143 patients with HPV16 DNA-negative tumours and missing value imputation.

Variable	Loco-regional tumour control		Overall survival	
	HR (95% CI)	*p*-value	HR (95% CI)	p-value
**Baseline model**				
Age (years)	0.94 (0.90–0.98)	**0.009**		
Oral cavity (yes vs no [b])	1.70 (0.76–3.82)	0.20	1.76 (1.03–3.01)	**0.038**
ECE status (1 vs 0 [b])			1.89 (1.08–3.31)	**0.025**
T stage (3,4 vs 1,2 [b])	2.21 (0.99–4.90)	0.053	1.82 (1.07–3.08)	**0.027**
*CD44*	0.50 (0.27–0.91)	**0.022**		
15-gene hypoxia classifier	3.55 (1.19–10.6)	**0.024**	1.48 (0.84–2.63)	0.18
C-Index (95% CI)	0.77 (0.67–0.86)		0.69 (0.62–0.76)	
**Extended model**				
Age (years)	0.95 (0.90–1.00)	0.058		
Oral cavity (yes vs no [b])	1.18 (0.36–3.85)	0.78	1.74 (1.00–3.01)	**0.050**
ECE status (1 vs 0 [b])			1.64 (0.90–3.00)	0.11
T stage (3,4 vs 1,2 [b])	1.10 (0.41–2.96)	0.85	1.31 (0.74–2.31)	0.35
*CD44*	0.71 (0.40–1.27)	0.25		
15-gene hypoxia classifier	2.45 (0.63–9.47)	0.19	1.16 (0.64–2.11)	0.63
HICR	3.03 (2.20–4.17)	**<0.001**	1.87 (1.55–2.25)	**<0.001**
7-gene signature	1.68 (0.99–2.85)	0.055		
5-miRNA signature			1.41 (0.98–2.03)	0.063
ATM rs4988023 (aa+cc [b] vs ac)			0.25 (0.10–0.63)	**0.003**
C-Index (95% CI)	0.93 (0.90–0.96)		0.83 (0.77–0.88)	
**Data-driven model**				
Age (years)	0.96 (0.91–1.01)	0.087		
ECE status (1 vs 0 [b])			1.74 (0.98–3.11)	0.061
*CD44*	0.60 (0.38–0.96)	**0.035**		
HICR	3.00 (2.19–4.11)	**<0.001**	1.81 (1.50–2.19)	**<0.001**
7-gene signature	1.91 (1.29–2.83)	**0.001**	1.39 (1.09–1.78)	**0.008**
5-miRNA signature			1.54 (1.06–2.22)	**0.022**
ATM rs4988023 (aa+cc [b] vs ac)			0.22 (0.09–0.56)	**0.001**
C-Index (95% CI)	0.94 (0.91–0.97)		0.82 (0.76–0.87)	

**Abbreviations:** HR, hazard ratio; 95% CI, 95% confidence interval; ECE, extracapsular extension; HICR, HPV-independent classifier of disease recurrence; [b], baseline class.

Next, we defined two additional biomarker signatures for each endpoint. The extended signatures contained the parameters of the baseline model as fixed parameters and additional biomarkers identified by the DKTK-ROG partner sites, while for the data-driven signatures, the baseline parameters were not fixed, Table 1. For the endpoint LRC, HICR and the 7-gene signature were additionally selected in the extended model, while for OS, HICR, the 5-miRNA signature, and the SNP rs4988023 were selected. Compared to the baseline model, the C-Index of the extended model increased to 0.93 (0.90–0.96) for LRC and to 0.83 (0.77–0.88) for OS. In the data-driven models, similar parameters than in the extended models were selected, while non-relevant parameters were excluded. Here, the C-Index was 0.94 (0.91–0.97) for LRC and 0.82 (0.76–0.87) for OS.

Fig. 1 shows patient stratifications into two subgroups obtained by the three signatures using the 67th percentile of the corresponding prognostic index for LRC and OS. The cut-offs are given in the figure caption. Overall, the 33% of patients with the highest predicted risk show significantly worse LRC and OS for all models (*p* < 0.001) with particularly poor outcome when stratified by the extended or the data-driven signature.

**Fig. 1:**
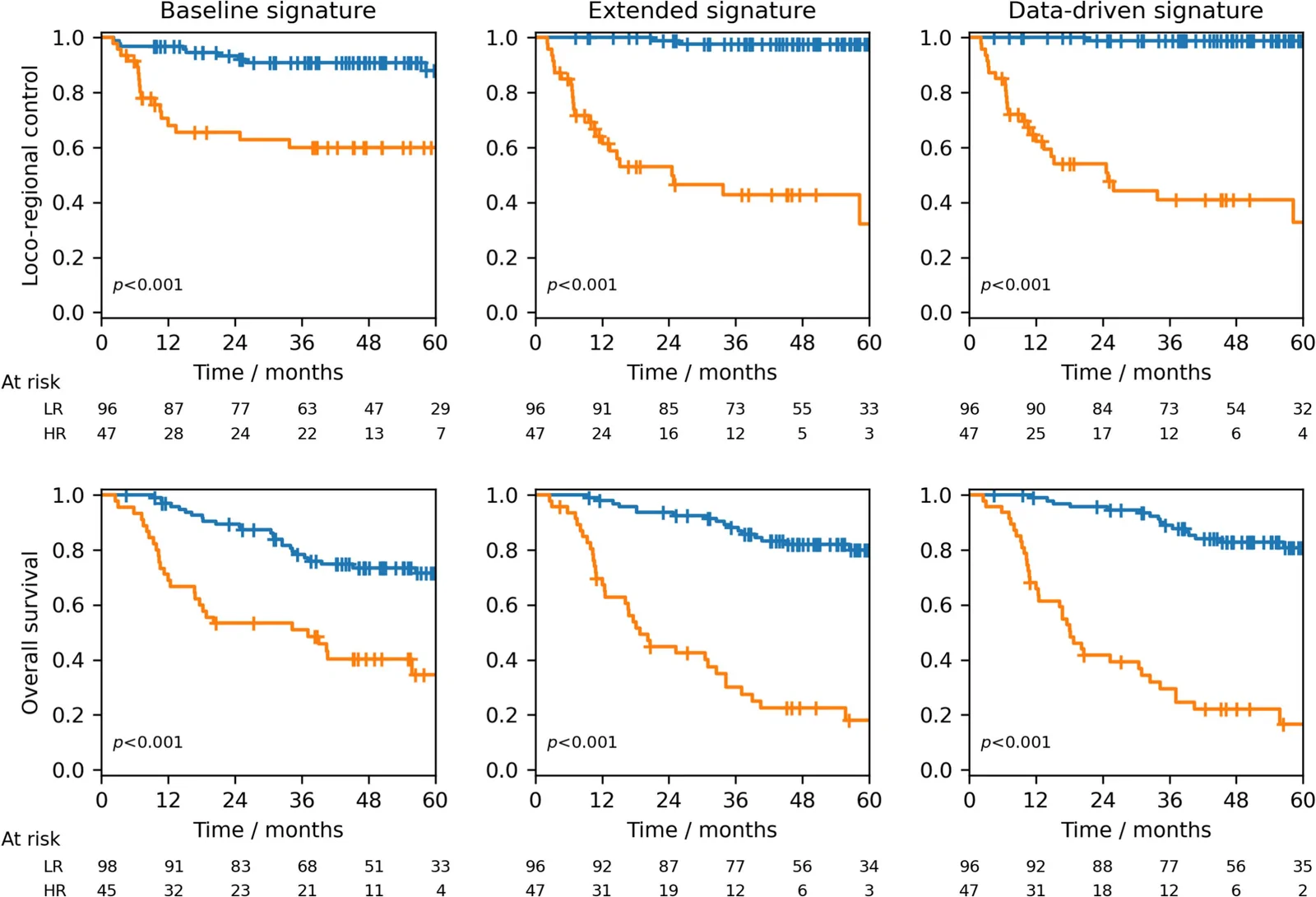
Kaplan-Meier estimates of loco-regional control (top, LRC) and overall survival (bottom, OS) for patients stratified into two risk groups based on the baseline biomarker signature (left), the extended biomarker signatures (centre), and the data-driven signature (right). LR: low risk (blue), HR: high risk (orange). The cut-offs were defined as follows: baseline signature: LRC: -1.621, OS: 1.235; extended signature: LRC: -2.793, OS: −0.277; data-driven signature: LRC: -3.170, OS: 0.0255. (For interpretation of the references to color in this figure legend, the reader is referred to the web version of this article.)

## 4 Discussion

In this manuscript, we pre-hoc defined potential biomarker profiles for upcoming validation within the completed prospective HNprädBio study. We combined different candidate biomarkers previously identified for patients with HPV16 DNA-negative HNSCC treated with PORT-C in nine centres of the DKTK-ROG [1]. Age, tumour location, T stage as well as the expression of the cancer stem cell-associated marker *CD44* and hypoxia had significant impact on LRC, and were combined in the baseline model. For the extended and data-driven models, we identified additional promising biomarkers or signatures, which improved the prognostic value for the endpoints LRC and OS. In particular, HICR, the 5-miRNA signature, and the 7-gene signature were important in most analyses for both endpoints.

The performance results reported in Table 1 and shown in Fig. 1, in particular for the extended and data-driven models with C-indices >0.9 for LRC and > 0.8 for OS, have to be regarded with caution. All three biomarker signatures included in these models (HICR, 5-miRNA, 7-gene signature) have been developed on the present retrospective DKTK-ROG cohort using elaborate machine-learning frameworks. Thus, they are at high risk for overfitting. In particular, these signatures have been independently validated previously, where they still showed significant but lower performance (e.g. C-index = 0.71 [13]), as expected. Thus, the reported performances in this manuscript may be overly optimistic. Furthermore, the strong association of these signatures with the endpoints may hinder the selection of other, less biased biomarkers and may assign disproportionate weight to these signatures. To assess the impact of these issues qualitatively, we repeated the analyses defining the extended and data-driven models with modified data. We added normally distributed random numbers of zero mean to the predictors of the three biomarker signatures to reduce their prognostic value. The standard deviations of the random numbers were chosen such that the C-Index decreased to approximately 0.7, which is more realistic and has been observed previously during validation of the 7-gene signature [13]. As a result, similar extended/data-driven signatures to those presented in Table 1 were obtained, with only minor variations, e.g., marker icSDF-1 was often included in addition. The overall stability observed across the models in this analysis underlines their potential robustness for the upcoming validation. Still, it may be required to re-fit or at least re-calibrate these models to the prospective HNprädBio dataset after locked prospective validation, as well as to confirm the univariable value of the individual markers.

The biomarkers in the baseline model, including the cancer stem cell-associated marker CD44 and the 15-gene hypoxia classifier have previously been shown to be prognostic for patients with locally advanced HNSCC who received surgery and PORT-C [11]. In addition, these markers were independently validated in a small monocentric, retrospective study [12]. Importantly, CD44 protein expression was also analysed using immunohistochemistry in these previous studies. Tumours with absent or low CD44 positivity showed 100% LRC [11], [12]. Therefore, this marker was not applicable for analysis with Cox regression in the present manuscript but is still of major interest for prospective validation.

The extended model includes the methylation-based classifier HICR, a 7-gene and a 5-miRNA signature, and the ATM SNP rs4988023, and therefore addresses additional biological aspects. The tumour DNA-methylome derived HICR signature contained 38 probes, including both hypo- and hypermethylated genes, some of which have been previously reported in different tumour entities [14]. For instance, the respective genes have been shown to lead to genetic instability (*TTC28* (a target of *TP53*) [25]), affecting T-cell differentiation (*EOMES* [26]), and to be associated with epithelial-to-mesenchymal transition (EMT), cell proliferation and invasion (*ESM-1* [27]). The 7-gene signature includes three individual genes (*HILPDA*, *CD24*, *TCF3*) and one metagene consisting of highly correlated genes (*ACTN1*, *INHBA*, *P4HA2*, *SERPINE1*) [13], which are for instance associated to hypoxia (*HILPDA* [28] and *P4HA2* [29]), cisplatin-resistance (*CD24* [30] and *SERPINE1* [31], [32]), cell proliferation and migration (*SERPINE1* [31], [32]), EMT (*INHBA* [33]) as well as self-renewal capacity of stem cells and resistance to ferroptosis (*TCF3* [34], [35]). The 5-miRNA signature incorporates miRNAs that are involved in several processes such as cell invasion and metastasis (hsa-let-7g [36]), hypoxic response, cell cycle regulation, DNA repair and immune response (hsa-miR-210 [37]), whereas for three of the miRNAs (hsa-miR-6508-5p, hsa-miR-4306, hsa-miR-7161-3p) no association to malignant processes has been reported to date [10]. ATM SNP rs4988023 is in complete linkage disequilibrium with the nonsynonymous ATM variant rs1801516 (r^2^ = 1) and has been used as a surrogate marker for this variant [38]. rs1801516 has previously been associated with cellular radiosensitivity in human fibroblasts [39], suggesting a potential link to radiation response, although its relevance to tumour radiosensitivity remains to be established.

Endpoint-specific associations may reflect different underlying determinants of outcome. LRC is more directly linked to tumour-intrinsic radiosensitivity and the local microenvironment, including stemness and hypoxia, whereas OS is additionally influenced, e.g. by distant progression, comorbidity and non-cancer mortality. Consequently, some biomarkers may preferentially associate with one endpoint.

As a limitation, as mentioned before, the multivariable results may be overly optimistic due to potential overfitting and model performance may be reduced when applied to the prospective dataset. Furthermore, missing value imputation may impact the results. The complete-case dataset across all considered biomarkers comprised only 76/143 patients and 17 of the 27 LRC events. Complete-case multivariable modelling would therefore have been severely constrained. Therefore, we used median/mode imputation as described above. The limited number of events relative to model complexity further increases the risk of overfitting, and the reported performance should be considered exploratory until prospective validation.

In conclusion, composite biomarker signatures were defined for patients with locally advanced HNSCC treated with PORT-C in a retrospective study of the DKTK-ROG. These signatures will now be validated in the prospective PORT-C HNSCC cohort of the HNprädBio trial of the DKTK-ROG that completed patient recruitment, before potential application in interventional clinical trials.

## Consent statement

The privacy rights of all human subjects have been observed and informed consent was obtained for retrospective data analysis from all human subjects.

## Ethics declaration

**Informed consent and patient details**: Written informed consent to take part in the study and to publish the article has been obtained from all participants or their legal representatives. The privacy rights of participants have been observed.

**Human and biological material**: This study included organ or tissue donors. This study includes human biological material and consent was obtained by donors, or their next of kin or legal representatives, for use in this study and for publication of the article. The samples used in this research were not sourced from executed prisoners or prisoners of conscience.

**Studies in Human**: This study was performed in compliance with relevant laws, regulatory frameworks and guidelines where the research took place. This study was approved by the Ethics Committee at TU Dresden (and of all other DKTK partner sites). (Approval No. EK 299092012)

## Declaration of competing interest

The authors declare the following financial interests/personal relationships which may be considered as potential competing interests: Michael Baumann, CEO and Scientific Chair of the German Cancer Research Center (DKFZ, Heidelberg) is responsible for collaborations with a large number of companies and institutions worldwide. In this capacity, he has signed contracts for research funding and/or collaborations, including commercial transfers, with industry and academia on behalf of his institute(s) and staff. He is a member of several supervisory boards, advisory boards and boards of trustees. Michael Baumann confirms that he has no conflict of interest with respect to this paper. In the past years, Mechthild Krause received funding for her research projects by Merck KGaA (2018-2020 for clinical study). Mechthild Krause and Annett Linge were involved in a publicly funded (German Federal Ministry of Education and Research) project with the companies Medipan (2019-2022), Attomol GmbH (2019-2022), GA Generic Assays GmbH (2019-2022), Gesellschaft für medizinische und wissenschaftliche genetische Analysen (2019-2022), Lipotype GmbH (2019-2022) and PolyAn GmbH (2019-2022). Mechthild Krause and Annett Linge confirm that, to the best of their knowledge, none of the above-mentioned funding sources were involved in the preparation of this paper. Simon Boeke reports conflicts of interest outside of the manuscript as follows: Merck (speaker fees, travel expenses), Elekta (speaker fees, travel expenses), Regeneron (speaker fees).
