# Supplementary material for: Definition of joint biomarker signatures for patients with locally advanced HNSCC treated by postoperative radiochemotherapy – A multicentre study of the DKTK-ROG
Source: Clin Transl Radiat Oncol. 2026 Oct 25;62:101330. doi: 10.1016/j.ctro.2026.101330 (PMC13737423; doi:10.1016/j.ctro.2026.101330)
Supplement: Supplementary file 1 [file mmc1.pdf]

Supplementary Table 1. Characteristics of the considered biomarkers for all 143 patients.

Variable			Median (range)
CD44			-0.07 (-2.77 – 1.80), missing: 13
SLC3A2			-2.78 (-4.27 – -1.28), missing: 13
HICR			-1.28 (-4.95 – 3.02), missing: 15
5-miRNA signature			0.04 (-3.17 – 3.01), missing: 58
7-gene signature			0.27 (-4.90 – 2.88), missing: 13
PD-L1-M			0.37 (0.00 – 0.99), missing: 51
	**Groups**	**Number**	**%**
15-gene hypoxia classifier	Less hypoxic/more hypoxic/missing	53/77/13	37.1/53.8/9.1
26-gene hypoxia classifier	Less hypoxic/more hypoxic/missing	36/94/13	25.2/65.7/9.1
PD-L1	Low/high/missing	69/31/43	48.3/21.7/30.1
CD8	Low/high/missing	73/27/43	51.0/18.9/30.1
icCXCR4	Negative/positive/missing	87/41/15	60.8/28.7/10.5
mCXCR4	Negative/positive/missing	116/12/15	81.1/8.4/10.5
icSDF-1	Negative/positive/missing	93/41/9	65.0/28.7/6.3
mSDF-1	Negative/positive/missing	123/11/9	86.0/7.7/6.3
Hypoxia-M	Less hypoxic/more hypoxic/missing	89/32/22	62.2/22.4/15.4
rs11615	cc/tc/tt/missing	17/67/45/14	11.9/46.9/31.5/9.8
rs13181	aa/ca/cc/missing	45/64/22/12	31.5/44.8/15.4/8.4
rs17655	cc/cg/gg/missing	5/53/73/12	3.5/37.1/51.0/8.4
rs25487	aa/ga/gg/missing	15/56/60/12	10.5/39.2/42.0/8.4
rs2267437	cc/cg/gg/missing	37/60/31/15	25.9/42.0/21.7/10.5
rs4988023	aa/ac/cc/missing	103/23/4	72.0/16.1/2.8/9.1

Supplementary Table 2. Univariable Cox regression of relevant clinical variables and the proposed biomarkers for loco-regional tumour control and overall survival including all available patients with HPV16-DNA negative tumours, without missing value imputation. HR = hazard ratio; 95% CI = 95 percent confidence interval.

	Loco-regional tumour control		Overall survival	
**Variable**	**HR (95% CI)**	**p-value**	**HR (95% CI)**	**p-value**
Sex (female vs male [b])	1.64 (0.72-3.75)	0.24	1.09 (0.59-2.03)	0.78
Age (years)	0.94 (0.90-0.99)	**0.010**	0.98 (0.95-1.01)	0.15
Dose (Gy)	1.08 (0.91-1.28)	0.39	1.10 (0.97-1.24)	0.14
Oral cavity (yes vs no [b])	2.51 (1.18-5.38)	**0.017**	2.10 (1.24-3.54)	**0.006**
ECE status (1 vs 0 [b])	1.58 (0.73-3.43)	0.25	2.02 (1.17-3.48)	**0.012**
T stage (3,4 vs 1,2 [b])	2.49 (1.15-5.39)	**0.021**	1.95 (1.16-3.29)	**0.012**
15-gene hypoxia classifier	4.70 (1.62-13.7)	**0.004**	1.88 (1.06-3.34)	**0.031**
26-gene hypoxia classifier	11.4 (1.55-84.3)	**0.017**	2.05 (1.03-4.08)	**0.040**
CD44	0.38 (0.22-0.68)	**<0.001**	0.82 (0.54-1.23)	0.33
SLC3A2	1.77 (0.95-3.30)	0.072	1.27 (0.84-1.93)	0.26
PD-L1	0.66 (0.24-1.81)	0.42	0.47 (0.22-1.00)	0.051
CD8	0.41 (0.12-1.41)	0.16	0.35 (0.15-0.84)	**0.019**
icCXCR4	1.67 (0.78-3.58)	0.18	1.11 (0.63-1.93)	0.72
mCXCR4	1.24 (0.37-4.14)	0.72	1.24 (0.53-2.90)	0.63
icSDF-1	2.53 (1.19-5.39)	**0.016**	1.11 (0.63-1.95)	0.73
mSDF-1	0.95 (0.22-4.00)	0.94	0.94 (0.34-2.60)	0.90
HICR	2.54 (1.99-3.25)	**<0.001**	1.83 (1.55-2.17)	**<0.001**
PD-L1-M	0.47 (0.11-1.98)	0.31	2.45 (0.08-0.73)	**0.011**
Hypoxia-M	1.18 (0.49-2.80)	0.71	1.98 (1.11-3.55)	**0.022**
5-miRNA signature	1.93 (1.24-2.99)	**0.003**	1.83 (1.34-2.50)	**<0.001**
7-gene signature	2.71 (1.84-4.00)	**<0.001**	1.69 (1.30-2.18)	**<0.001**
rs11615 (aa vs ca vs cc)	-	0.94	-	0.32
rs13181 (aa vs ca vs cc)	-	0.28	-	0.12
rs13181 (aa+ca [b] vs cc)	0.52 (0.16-1.73)	0.29	0.41 (0.16-1.04)	0.061
rs17655 (aa vs ca vs cc)	-	0.85	-	0.091
rs25487 (aa vs ca vs cc)	-	0.95	-	0.22
rs2267437 (aa vs ca vs cc)	-	0.24	-	0.19
rs4988023 (aa vs ac vs cc)	-	0.13	-	**0.023**
rs4988023 (aa+cc [b] vs ac)	0.67 (0.23-1.94)	0.46	0.46 (1.94-1.07)	0.070

Supplementary Table 3. Summary of methods for biomarker analyses and definition of biomarker risk variables.

Biomarker	Analysis method	Definition of risk
HPV16 DNA	PCR-array	
CD44	Real-time PCR	expression
SLC3A2	nanoString analysis	expression
15-gene hypoxia classifier	nanoString analysis	k-means clustering with 2 cluster centres
26-gene hypoxia classifier	nanoString analysis	k-means clustering with 2 cluster centres
HICR	Methylation profiling using Illumina microarrays	Risk score
5-miRNA signature	miRNA expression using SurePrint microarrays	Linear predictor
7-gene signature	nanoString analysis	Linear predictor
icCXCR4	Immunofluorescence	Staining intensity ≥2 in at least one core
mCXCR4		
icSDF-1		
mSDF-1		
CD8	Immunohistochemistry	sum score ≥6
PD-L1		
rs4988023	Real-time PCR	aa+cc vs ac

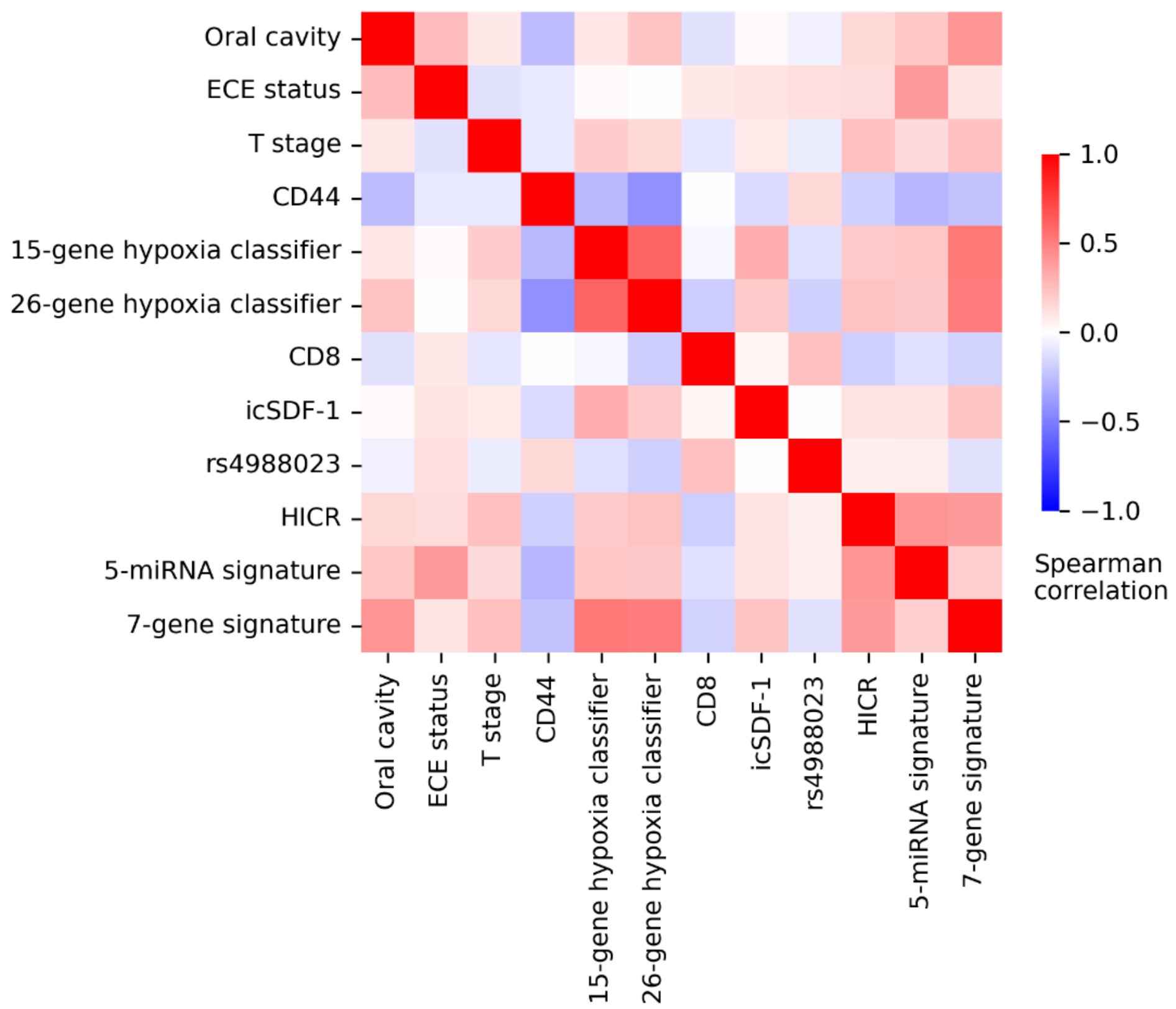

Supplementary Figure 1. Spearman correlation between biomarkers with significant association to loco-regional control (LRC) or overall survival (OS).
